# Horizons of Psychiatric Genetics and Epigenetics: Where Are We and Where Are We Heading?

**Published:** 2014

**Authors:** Hamid Mostafavi Abdolmaleky

**Affiliations:** 1 Assistant Professor, Department of Psychiatry, Iran University of Medical Sciences, Tehran, Iran AND Research Associate, Department of Genetics and Genomics, School of Medicine, Boston University, Boston, MA, USA

## Abstract

Today multinational studies using genome-wide association scan (GWAS) for >1000,000 polymorphisms on >100,000 cases with major psychiatric diseases versus controls, combined with next-generation sequencing have found ~100 genetic polymorphisms associated with schizophrenia (SCZ), bipolar disorder (BD), autism, attention deficit and hyperactivity disorder (ADHD), etc. However, the effect size of each genetic mutation has been generally low (<1%), and altogether could portray a tiny fraction of these mental diseases. Furthermore, none of these polymorphisms was specific to disease phenotypes indicating that they are simply genetic risk factors rather than causal mutations.

The lack of identification of the major gene(s) in huge genetic studies increased the tendency for reexamining the roles of environmental factors in psychiatric and other complex diseases. However, this time at cellular/molecular levels mediated by epigenetic mechanisms that are heritable, but reversible while interacting with the environment. Now, gene-specific or whole-genome epigenetic analyses have introduced hundreds of aberrant epigenetic marks in the blood or brain of individuals with psychiatric diseases that include aberrations in DNA methylation, histone modifications and microRNA expression. Interestingly, most of the current psychiatric drugs such as valproate, lithium, antidepressants, antipsychotics and even electroconvulsive therapy (ECT) modulate epigenetic codes.

The existing data indicate that, the impacts of environment/nurture, including the uterine milieu and early-life events might be more significant than genetic/nature in most psychiatric diseases. The lack of significant results in large-scale genetic studies led to revise the bolded roles of genetics and now we are at the turning point of genomics for reconsidering environmental factors that through epigenetic mechanisms may impact the brain development/functions causing disease phenotypes.

**Psychiatric Genetic:Accomplishments and Challenges**

The advancement of genetic sciences and the development of new techniques greatly improved our understanding of mental diseases during the last decade and this is going to open new windows toward the diagnosis and treatment of psychiatric diseases in coming years. While ten years ago genetic studies were limited to single gene analysis in hundreds of cases and controls, today multinational consortiums have executed GWAS for >1000,000 known polymorphisms (mutations) on hundreds of thousands of cases and controls affected by major psychiatric diseases. They found hundreds of genetic polymorphisms or copy number variations (CNVs) (due to deletion or insertion) associated with SCZ and BD, autism and ADHD, many of which were also identified by smaller traditional genetic association and family studies. Interestingly, several of these polymorphisms are shared by different mental diseases (1), particularly in SCZ and BD (2, 3). Some important shared genes include CACNA1C (α-1C subunit of the L-type voltage-gated calcium channel), ZNF804A, PBRM1, neurogranin, SYNE1 and major histocompatibility region on chromosome 6 (4-7).

Despite this progress, the effect size of each genetic mutation (which most of them are intergenic), including CNVs and de novo mutations, has been generally low (<1%), and altogether could portray a tiny fraction of these mental diseases (3, 8). Some investigators suggest that, this small effect size could be due to the heterogeneity of mental diseases. Thus more sample sizes are required (e.g. >100,000). However, doubling the sample size during the last five years, combined with new analytical methods, could just increase the number of candidate genes, including those affected by de novo mutations, still with small effect size (9, 10). Notably, unlike known genetic diseases such as Huntington’s Disease or thalassemia, none of these polymorphisms were specific to the disease phenotypes, including endophenotypes (11), and were only more frequent in disease state, compared to the controls. This indicates that these polymorphisms can be simply considered as risk factors, and there could not be ~100 types of SCZ or BD matching to ~100 identified genes. Hence, the actual cause(s) should be other players to be identified.

The GWAS findings in major depressive disorder (MDD) and obsessive-compulsive disorder (OCD) have been even more frustrating as no specific gene could surpass the threshold of significant association (12, 13), even in large studies with almost ten thousand cases of MDD versus controls (14). Although the development of next generation sequencing technology (the most advanced method for the detection of unknown mutations in whole exome/genome analysis) could help to identify a number of new candidate genes for SCZ (e.g. GRM5, PPEF2 and LRP1B), and autism (e.g. SCN2A), it has yet to introduce any gene(s) with a key role in major mental diseases (15-21).

While, these large-scale genetic studies have not been successful to tease out the main underlying genetic causes of major mental diseases, several pharmacogenomic studies support that specific genetic polymorphisms can predict the side effects and probability of responsiveness to specific psychiatric drugs (22-27). Hence, a number of companies have started to utilize a smaller set of genetic data to establish personalized treatment protocols for individuals affected by major mental diseases (e.g.www.genomind.com and http://genelex.com). This new approach for personalized medicine is going to be complemented with the induced pluripotent stem (iPS) cell technology that can generate iPS cells from the peripheral cells of affected individuals containing all genetic mutations of the original cells. These cells can be differentiated to neuronal cells in culture (e.g. dopaminergic, serotoninergic or GABAergic cells) and the effect of the candidate drugs can be evaluated in a Petri dish (28), like an antibiogram.

**Psychiatric Epigenetics: Achievements and Opportunities**

The lack of identification of major gene(s) linked to psychiatric diseases in GWAS and deeper analyses using next generation sequencing technology, increased the tendency of scientific communities for reexamining the roles of environmental factors in impacting the brain development and functions. However, this time at cellular or molecular levels mediated by latterly discovered epigenetic mechanisms. Epigenetics refers to the science of complementary chromosomal codes (beside genetic codes) that govern cellular differentiations and regulate genes expression in a dynamic manner based on the tissue types, developmental periods and micro/macro-environmental conditions (such as hormonal effects, cell metabolic state, nutritional habits/status, seasonal or ecological conditions). So far, four major mechanisms have been introduced for epigenetic regulations, including DNA methylation, histone modifications, the interference of non-coding microRNAs and RNA editing (29).

Well-known varieties of DNA methylation include methylation of cytosine nucleotides that are followed by guanine or adenine, and hydroxymethylation that is a temporary product during the conversion of methylated cytosines to unmethylated cytosines. Unlike other tissues, hydroxymethylation is quite abundant in the human brain. While DNA methylation generally suppresses gene expression, hydroxymethylation can induce gene expression and appears to play a key role in functional plasticity of neuronal cells that barely replicate. While several types of DNA methyltransferase enzymes induce DNA methylation, TET and IDH family of enzymes catalyze hydroxymethylation.

Histone modifications which comprise acetylation, methylation, ubiquitination, etc. of different amino acids of histone proteins may suppress or increase the expression of interconnected genes depend on the identity and location of those amino acids. Several types of enzymes such as histone acetylases, histone deacetylases (HDACs) (that is inhibited by valproate), histone methylases and demethylases are involved in histone modifications (30).

DNA methylation and histone modifications have complex interplays with more than 1000 recently discovered non-coding microRNAs (~20 bases in length) that can each target over 100 genes, even in other tissues (i.e., exosomal or circulating miRNAs). miRNAs generally suppress RNA expression or degrade the transcribed RNA, thus inhibit protein synthesis. RNA editing that is engaged in diverse splicing of the RNA transcripts (generating multiple isoforms of protein with functional diversities) makes epigenetic regulation even more complex.

During the last ten years, several epigenetic studies, using gene-specific or whole genome epigenetic analysis (e.g. the Illumina 27 k or 450 k DNA methylation array, and next generation sequencing following Immunoprecipitation of methylated DNA or acetylated/methylated chromatin) have introduced hundreds of aberrant epigenetic marks in the blood or brain of individuals with psychiatric diseases. Examples include aberrant DNA methylation of RELN, MB-COMT, HTR2A, ST6GALNAC1, AKT1, DNMT1, DTNBP1, NOS1, PPP3CC and 5-HTT in brain (31-36) and COMTD1, HTR1E, CD224, CD7, LAX1, MPG, MPO, PRF1, TXK, FAM63B and RELN in the blood (37-39) of patients with SCZ and/or BD, and AFF2, GABRB3, JMJD1C, KCNJ10 , NLGN2, SNRPN, SNURF, PIK3C3 and UBE3A in the blood of autistic patients (40).

In the field of histone modifications, most of the studies have been related to SCZ and BD providing strong evidence for aberrant expression of several genes of histone proteins, including HIST1H2BC, HIST1H2BD, HIST1H2BH, HIST1H2BG, HIST1H4K and HIST2H2BE) in the blood cells of these patients and/or their first degree relatives (41, 42). Additionally, there are reports indicating i) an increase in the expression of H3-(methyl)arginine 17 in the prefrontal cortex associated with decreases in the expression of CRYM, MDH, OAT and CYTOC/CYC1 which are considered as metabolic genes (43), ii) aberrant expression of enzymes such as HDAC3 in the temporal cortex, which removes acetyl groups from the histone proteins decreasing gene expression (44), iii) an increased expression of HDAC1 in the frontal cortex, and an inverse association between the expression of HDAC1, HDAC3 or HDAC4 and GAD67 expression in SCZ patients (45).

Findings related to the roles of miRNA in mental disorders are also promising. Examples include, increased expression of miR15a and b, miR195, miR181b, miR107, exosomal miR29c and miR497 (46, 47) and decreased expression of miR24, miR26b, miR30e miR92 and miR346 (48) in the post-mortem brains of SCZ and/or BD patients. A decrease in the amount of circulating miR134 has been shown in the blood of BD patients in a manic phase, as well (49). In the blood of autistic patients increased expression of miR23a&b, miR132, miR146a and b, miR663,miR29b and miR103 and decreased expression of miR92a1/2, miR320, miR363, miR139-5p and miR219-5p were uncovered in miRNA microarray analysis (50, 51). However, the underlying origin of miRNA dysregulations as well as other epigenetic aberrations remained to be identified for preventive or therapeutic interventions.

Although hundreds of statistically significant genetic and/or epigenetic alterations have been found in major mental diseases, most of the epigenetic and even genetic alterations by themselves do not necessarily lead to disease phenotype. In fact, they should induce biologically significant deleterious changes in the expression of coding genes or the structure of proteins to lead to the disease state. Considering the functional impacts of genetic/epigenetic alterations, at least at expression level, more than two dozen whole genome transcriptome analyses have taken place introducing hundreds of neuronal, synaptic and inflammatory genes with aberrant expression in the brain and/or blood of patients with psychiatric diseases (41, 52). Correlation of gene-specific or genome-wide epigenetic alterations with the expression of corresponding genes could identify epigenetic mechanisms of the altered functionality of more than a dozen genes related to synaptic transmission and axonal guidance (e.g. MB-COMT, HTR2A, 5-HTT, dopamine receptors, GAD1, RELN, DTNBP1, HTR1E, NOS1, PPP3CC, GRM5, PRIMA1, SHANK3) in major mental diseases, including autism (31-33, 36, 53-57).

It is important to note that, whereas some of these alterations might be tolerable in normal life, they may fail to adjust with specific conditions such as stressful life or exposure to contaminants and/or malnutrition. Hence, the dynamics of interactions between genome/epigenome and environment factors makes the issue more complex, a problem that in part can be assessed in a Petri dish using iPS cells of the affected or at risk individuals. Additionally, since epigenetic marks are mainly tissue-specific and blood epigenome cannot generally portray the brain epigenetic landscape, a number of studies undertook both brain and blood or saliva epigenetic analysis to identify peripheral epigenetic marks that represent the brain alterations. Dempster et al., found epigenetic aberrations of ST6GALNAC1 both in blood and brain of patients with psychosis (34). Others identified the same epigenetic alterations of MB-COMT, HTR2A and 5-HTT in brain and DNA extracted from the saliva of patients with SCZ or BD (36, 58, 59). Until now, these studies have not been undertaken in the same individuals using an adequate sample size, due to ethical issues associated with the brain biopsy in living individuals. Therefore, this important line of research remains to be accomplished using novel approaches in coming years. Certainly, the detection of peripheral biomarkers mirroring the inaccessible brain tissues can revolutionize psychiatric evaluations with diagnostic, preventive or therapeutic applications.

**Psychiatric Drugs and Epigenetic Modifications**

Several studies have shown that many current psychiatric drugs are, in fact, epigenetic modifiers. For example, valproate is a well-known HDAC inhibitor, and increases acetylated histone inducing gene expression, particularly in patients with BD (60). Lithium can also increase histone acetylation (61) and decrease global DNA methylation in patients who respond to this drug (62). Most of the antipsychotic drugs such as benzamides, clozapine, and lurasidone (63-65) and different classes of antidepressants (66-69) as well as ECT (70) are also epigenetic modifiers. There are also extensive efforts to identify novel epigenetic drugs that could target the affected genes/pathways for preventive of therapeutic remedies. While these efforts promise the development of novel drugs in near future, the lack of tissue specificity of most drugs remains a dilemma, not only in psychiatry, but also all branches of medicine which require to be addressed in coming years. Although new techniques such as TALEN and CRISPR are successfully used in laboratory experiments for mutational repairs and the delivery of epigenetic modifiers to specific genomic regions to modulate genes expression, these techniques are not likely to have clinical applications in the current decade.

## Conclusion

The fact that the same genetic mutations (1, 4, 7, 8) or epigenetic aberrations (epimutations), are linked to the pathogenesis of several psychiatric diseases is among the most interesting findings of recent GWAS and epigenetic analyses that support pleiotropic functions of these genes. Pleiotropy means that a specific gene may have several functions in different ages and/or tissues. Hence, its malfunction may present various manifestations in different ages/tissues. For example, Tourette syndrome, ADHD, OCD, anxiety disorders and depression are closely linked together, so that the affected individual may show ADHD before tics in childhood, OCD in adolescence and anxiety and/or depression in adulthood (71). Therefore, a single drug may be appropriate for the adjustment of the activity of a pleiotropic gene improving all diverse phenotypes of that genetic/epigenetic disease in different ages or tissues.

Based on the studies reviewed here, the role of environment/nurture, including the uterine milieu might be more significant than genetic/nature in the genesis of most psychiatric diseases. While the normal epigenetic landscape is mostly established in the fetal period, followed by childhood and adulthood periods (72), several lines of evidence indicate that early life environmental impacts can alter epigenetic landscape with life-long effects. For examples, there are significant changes in DNA methylation profile of blood cells obtained from the umbilical cord of infants with arsenic exposure in utero (73). Furthermore, various factors such as nutritional imbalance, stress, smoking, drug abuse, birth weight and even seasonal changes in maternal diet during the periconceptional period may hamper the normal establishment of the epigeneme (74-81). More importantly, many studies provided strong experimental evidence that the acquired epigenetic alterations can be retransferred to the next generations (82, 83) mimicking the inheritance of genetic diseases. Therefore, the shared environment of family members dictating the shared epigenetic portraits might be the origin of higher rate of familial psychiatric disorders misinterpreted as genetic diseases. Notably, even monozygotic twins with almost 100% genetic similarity only show ~45% concordance rate in SCZ that better fit with the idea of shared generational milieu, including uterine environment and maternal nutritional habits rather than genetic mutations.

The results of extensive genetic analyses during the last two decades, directing the scientific community to rethink and revise the bolded roles of genetic factors in psychiatric and other complex diseases. Indeed, we are at the turning point of the age of genomics for reconsidering environmental factors that through epigenetic mechanisms may impact the brain development and functions at cellular or molecular levels. While epigenetic modifications may also balance the malfunction of genetic mutations, the ongoing deeper genetic/epigenetic analyses using the technology of next generation sequencing may help to recognize the combined genetic and epigenetic alterations leading to disease state with diagnostic, preventive and therapeutic applications in coming years. 

## Notes

**Declaration of interest:** None.
